# On the Multivariate Extremal Index

**DOI:** 10.6028/jres.099.052

**Published:** 1994

**Authors:** S. Nandagopalan

**Affiliations:** Colorado State University, Fort Collins, CO 80523

**Keywords:** dependence function, exceedance, extremal index, multivariate, point process, stationary

## Abstract

The exceedance point process approach of Hsing et al. is extended to multivariate stationary sequences and some weak convergence results are obtained. It is well known that under general mixing assumptions, high level exceedances typically have a limiting Compound Poisson structure where multiple events are caused by the clustering of exceedances. In this paper we explore (a) the precise effect of such clustering on the limit, and (b) the relationship between point process convergence and the limiting behavior of maxima. Following this, the notion of multivariate extremal index is introduced which is shown to have properties analogous to its univariate counterpart. Two examples of bivariate moving average sequences are presented for which the extremal index is calculated in some special cases.

## 1. Introduction

Extreme value theory for multivariate iid sequences has been studied for quite some time now but attention to the dependent case has been relatively recent. For univariate sequences it is known that local dependence causes extreme values to occur in clusters, which in turn results in a stochastically smaller distribution for the maximum than if the observations were independent. We begin with a brief review of these results, which we shall later extend to the multivariate case.

Let {*ξ_n_*} be a univariate stationary sequence. Write *M_n_* = max{*ξ*_1_,…,*ξ_n_*} and for τ>0, let {*u_n_*(τ)} denote a sequence satisfying lim*_n_*_→_*_∞_np*{*ξ*_1_>*u_n_*(τ)}=τ. Under quite general mixing assumptions there exist constants 0≤ *θ*′≤ *θ*″≤1 such that

$$\begin{array}{c} \underset{n\to \infty }{\lim\;\sup}\;P\left\{M_{n}\leq u_{n}\left(\tau \right)\right\}=e^{\theta \tau }\;\text{and}\\ \underset{n\to \infty }{\lim\;\inf}\;P\left\{M_{n}\leq u_{n}\left(\tau \right)\right\}=e^{\theta^{\prime \prime }\tau } \end{array}$$

for all τ. (See Ref. [1], although the idea actually dates back to Refs. [2–4].) Thus if *P*{*M_n_*≤*u_n_*(τ_0_)} converges for some τ_0_, then *θ*′=*θ*″(=*θ*, say) and hence lim*_n_*_→∞_ *P*{*M_n_*≤*u_n_* (τ)}=c^−^*^θ^*^r^ for all τ>0. The common value *θ* is then called the *extremal index* of {*ξ_n_*}. We shall assume *θ* to be positive whenever it exists, since the case *θ*=0 corresponds to a degenerate limiting distribution for *M_n_*. Note that *θ*=1 for iid sequences. Let $\left\{{\hat{\xi }}_{n}\right\}$ be an iid sequence with ${\hat{\xi }}_{1}{=}^{d}\xi_{1}$, called the *associated iid sequence*, and write ${\hat{M}}_{n}=\max\left\{{\hat{\xi }}_{1},\ldots ,{\hat{\xi }}_{n}\right\}$. If {*ξ_n_*} has extremal index *θ* and lim*_n_*_→∞_ *P*{*M_n_*≤*v_n_*(*t*)} = *H*(*t*) for a suitable family of normalizing constants {*v_n_*(*t*)}, then it follows (upon identifying e^−^*^θ^*^r^ with *H*(*t*)) that $\lim_{n\to \infty }P\left\{{\hat{M}}_{n}\leq v_{n}\left(t\right)\right\}=\hat{H}\left(t\right)$where

$$H\left(t\right)=\hat{H}{\left(t\right)}^{\theta }.$$ (1)

The extremal index is thus a measure of the effect of dependence on the limiting distribution of *M_n_*. The stochastically smaller limiting distribution of *M_n_* is in fact a direct result of the clustering of extremes, as explained below. See Ref. [5] for details.

For fixed *τ*>0 let the *exceedance point process* $N_{n}=N_{n}^{\left(r\right)}$ be defined by

$$\begin{array}{ll} N_{n}\left(B\right)=\sum_{i=1}^{n}l_{\left(\xi_{n}>n_{n}\left(\tau \right)\right)}l_{\left\{l\;n\in B\right\}}, & B\subset \left[0,1\right], \end{array}$$

where *I_A_* denotes the indicator function of the event *A*. Then for a broad class of weakly dependent sequences, the limit in distribution of *N_n_*, if it exists, is a Compound Poisson process with intensity *θ*τ and multiplicity distribution π on {1,2,…}. The Poisson events may in fact be regarded as the positions of “cxceedance clusters” while the multiplicities correspond to cluster sizes. More explicitly, one may divide the *n* observations into *k_n_* blocks of roughly equal size and regard exceedances within each block as forming a single “cluster”, so that the cluster sizes are given by *N_n_*(*J_i_*), *i=l*,…,*k_n_*,where $J_{r}\left(=J_{n,t}\right)=\left(\frac{i-1}{k_{n}},\;\frac{i}{k_{n}}\right]$. For a suitable choice of *k_n_* depending on the mixing rate of {*ξ_n_*}, one then has

$$\begin{array}{ll} \underset{n\to \infty }{\lim}P\left\{N_{n}\left(J_{1}\right)=j|N_{n}\left(J_{1}\right)>0\right\}=n\left(j\right). & j\geq 1. \end{array}$$

and

$$\begin{array}{c} \underset{n\to \infty }{\lim}P^{k_{n}}\left\{N_{n}\left(J_{1}\right)=0\right\}=\underset{n\to \infty }{\lim}P\left\{N_{n}\left[0,\;1\right]\;=0\right\}\\ =\underset{n\to \infty }{\lim}P\left\{M_{n}\leq u_{n}\left(\tau \right)\right\}=c^{\theta \tau }, \end{array}$$

so that in particular, lim*_n_*_→∞_ *k_n_ P*{*N_n_*(*J*_t_)>0}=*θ*τ. Hence

$$\begin{array}{c} \underset{n\to \infty }{\lim}E\;N_{n}\left[0,1\right]=\underset{n\to \infty }{\lim}k_{n}F\;N_{n}\left(J_{1}\right)\\ \underset{n\to \infty }{\lim}k_{n}E\left(N_{n}\left(J_{1}\right)|N_{n}\left(J_{1}\right)>0)P\left\{N_{n}\left(J_{1}\right)>0\right\}\right)\\ =\theta \tau \;\underset{n\to \infty }{\lim}\;E\left(N_{n}\left(J_{1}\right)|N_{n}\left(J_{1}\right)>0\right), \end{array}$$

while on the other hand, lint_n→∞_ *EN_n_*[0,1]=lim*_n_*_→∞_ *nP*{*ξ*_1_>*u_n_*(τ)}=*τ*. The cluster size distribution and the extremal index are therefore related by

$$\underset{n\to \infty }{\lim}\;E\left(N_{n}\left(J_{1}\right)|N_{n}\left(J_{1}\right)>0\right)=1/\theta .$$ (2)

Now let {*ξ_n_*=(*ξ_nl_*,…,*ξ_nd_*), n∈ℤ} be a multivariate stationary sequence where *d*≥1 is a fixed integer, and write *M_n_*=(*M_nl_*,…, *M_nd_*) where *M_nj_* = max{*ξ_ni_*,…,*ξ_nj_*}, *j*−1,…,*d*. The study of multivariate extremes began in the early 1950s, focusing mainly on the limiting behavior of *M_n_* under a linear normalization, when the observations are iid. The resulting class of limiting distributions was characterized in Ref. [6] and domains of attraction criteria were given in Ref. [7]. See also Ref. [8]. Chapter 5, for an account of the literature surrounding this theory. For stationary sequences satisfying a general mixing assumption, it is known (see Refs. [9, 10], and Theorem 1.1 below) that the class of limiting distributions of *M_n_* is the same as for iid sequences. In this paper we explore the precise effect of dependence on the limiting distribution by extending the univariate theory described above to the multivariate case. Essentially, this involves studying the inter-relationship between the two dependence structures present, one due to dependence over time and the other due to the dependence between the various components of the multivariate observations. The ideas become most transparent when presented in terms of so-called dependence functions [8]. Here we adopt the slightly modified definition found in Ref. [9]. A distribution function *D* on [0,1]*^d^* it called a *dependence function* if *D_j_*(*D_j_*{*u*))=*D_l_*(*u*), *u*∈|0,1|, *j*=1,…,*d*, where the subscript *j* signifies they *j*th marginal. The dependence function of a distribution *F* on IR*^d^* is defined by D*_f_*(*u*)=*P*{F_1_(*X*_1_)≤ u*_l_*,…,*F_d_*(*X_d_*)≤*u_d_*}, *u*=(*u_l_*,…,*u_d_*)∈ [0,1]*^d^*, where (*X*_1_,…,*X_d_*) is a random vector with distribution *F*. More generally, any dependence function satisfying *F*(*x*)=D(*F*_1_(*x*_1_),*…*,*F_d_*(*X_d_*)) could be defined to be a dependence function of *F*, although the present choice is a natural one.

Write *T*=(0,1)*^d^\*{l} where l=(l,…,1)∈IR*^d^*, and for *t*=(*t*_l_,…,*t_d_*)∈*T*, let *v_n_*(*t*)=(*v_nl_*(*t_l_*),…,*v_nd_*(*t_d_*)) where *v_nj_*(*t_j_*) satisfies lim*_n_*_→∞_ *nP*{*ξ_lj_*>*v_nj_*(*t_j_*)}=− log*t_j_*. Let *H_n_*denote the distribution function of *M_n_*(i.e., *H_n_*(*x*)=*P*{*M_n_*≤*x*}), with marginals *H_nj_*, *j*=l,…,*d*. Then (see Refs. [8, 11]).

$$H_{n}\left(v_{n}\left(t\right)\right){\to^{\prime }}^{\prime }\;H\left(t\right)$$ (3)

if and only if

$$H_{nj}\left(v_{nj}\left(t_{j}\right)\right)\to^{H}\;H_{t}\left(t_{j}\right),j=1,\ldots ,d,\;\text{and}\;D_{jtn}\left(t\right)\to^{n}D_{H}\left(t\right).$$

The limiting behavior of *M_n_* can therefore be separated into two parts, one pertaining to the convergence of the marginals (a univariate problem) and the other to the convergence of the dependence functions. Here we focus attention exclusively on the latter. It should be noted that the choice of normalising constants does not affect the dependence function of the limit distribution *H*, but only alters the marginals (sec Ref. [9], Lemma 3.2). Since our main interest is in the dependence function, the present choice of normalising constants is appropriate in view of the fact that it results in Uniform[0,1] marginals for the limit distribution when {*ξ_n_*} is iid, so that in particular *D_H_*=*H*. According to Theorem 3.3 of Ref. [9], the class of all possible limits *H* in Eq. (3) (for iid {*ξ_n_*}) is precisely the class of *extreme* dependence functions, that is those that satisfy

$$D^{n}\left(t\right)=D\left(t_{1}^{n},\ldots ,t_{d}^{n}\right)$$ (4)

for each *n*>1 and t=(*t_l_*,…,*t_d_*)∈[0,1]*^d^*. Theorem 1.1 below shows that the same is true also if {*ξ_n_*} is a stationary sequence satisfying the following mixing condition.

For t∈*T*, let

$${ℬ}_{k}^{1}\left(v_{n}\left(t\right)\right)=\sigma \left\{\left(\xi_{ij}>v_{nj}\left(t_{j}\right)\right):k\leq i\leq l,\;j=1,\ldots ,d\right\}$$

and for 1≤*l*≤*n*−1 define

$$\begin{array}{c} \alpha_{n,l}=\sup\{\left|P\left(A\cap B\right)-P\left(A\right)P\left(B\right)\right|:A\in {ℬ}_{1}^{k}\left(v_{n}\left(t\right)\right),\\ B\in {ℬ}_{k+l}^{n}\left(v_{n}\left(t\right)\right),1\leq k<k+l\leq n\}. \end{array}$$

The mixing condition Δ(*v_n_*(t}) is then said to hold if $\alpha_{n,I_{n}}\to 0$ for some sequence {*l_n_*} satisfying *l_n_ln*→0. This is the multivariate version of the mixing condition used in Ref. [5] and is slightly stronger than the *D*(*u_n_*) condition in Ref. [9]. Henceforth {*ξ_n_*} will be assumed to satisfy Δ(*v_n_*(t)), for some or all **t**, as required.

Theorem 1.1. *Let* {*ξ_n_*} *satisfy* Δ(*v_n_*(t)) *for all* **t**∈*T and suppose that P*{*M_n_*≤*v_n_*(**t**))→*^H^ H*(**t**), *non-degenerate. Then D_n_ is an extreme dependence function and hence*, *in particular*, *H*(**t***^c^*)=*H^c^*(**t**) *for each* **t**∈[0.1]*^d^ and c*>0 (*where* $t^{c}=\left(t_{1}^{c},\ldots ,t_{d}^{c}\right)$).

Proof: The first part is an immediate consequence of Theorem 4.2 of Ref. [9] while the second part follows from the definition of extreme dependence functions upon noting that (by the univariate theory described above), the marginals of *H* are of the form $H_{j}\left(t_{j}\right)=t_{j}^{\theta_{j}}$where *θ_j_* is the extremal index of {*ξ_nj_*}, the *j*th-component sequence of {*ξ_n_*}.

In the next section we apply the exceedance point process approach to multivariate extremes and obtain some weak convergence results. The multivariate extremal index is then defined (in Sec. 3), based on the multivariate analogue of Eq. (1). It is seen to be a function of only *d* − 1 variables and its properties naturally extend those of the univariate extremal index. Finally in Sec. 4 we consider two examples of bivariate moving average sequences for which the computation of the extremal index is demonstrated.

## 2. Exceedance Point Processes

Fix *t*∈*T* and let $\delta_{ij}=I_{\{\xi_{ij}>\nu_{nj}\left(\tau_{j}\right)\},}\;i=1,\ldots ,n,\;j=1,\ldots d$, and put δ*_i_*=(δ*_i_*_l_,…,δ*_id_*). The *multivariate exceedance point process* $N_{n}=N_{n}^{\left(t\right)}$ is then defined by

$$\begin{array}{cc} N_{n}\left(B\right)=\sum_{i=1}^{n}I_{\left(itn\in B\right)}\delta_{1}, & B\in \left[0,1\right]. \end{array}$$ (5)

Assume that {*ξ_n_*} satisfies Δ(*v_n_*(**t**)). If also *N_n_*→*^d^ N*_0_, then it may be shown (as in the univariate case) that the limit *N*_0_ is a point process on [0,1] which is of Compound Poisson type. More precisely, the Laplace Transform of *N*_0_ is given by

$$\begin{array}{c} -\log E\;\exp\left\{-\sum_{j=1}^{d}\int_{\left|0,1\right|}f_{j}\;dN_{0j}\;\right\}=v\int_{x\in \left|0,1\right|}\int_{y\in x_{4}^{d+}}\;\\ \left(1-\exp\left\{-\sum_{j=1}^{d}yf_{j}\left(x\right)\right\}\right)d\pi \left(y\right)dx. \end{array}$$ (6)

Here *N*_0_*_j_* denotes the *j*th-component of *N*_0,_ *v* is a positive constant, π is a probability distribution on ${\mathbb{Z}}_{+}^{d1}={\left\{0,1,2,\ldots \right\}}^{d}\backslash \left\{0\right\}$ and *f_j_*’s are non-negative functions on [0,1].

Let {*k_n_*} be any sequence of positive integers satisfying

$$\begin{array}{cc} k_{n}\to \infty ,\;k_{n}l_{n}/n\to 0,\;\text{and}\;k_{n}\alpha_{n\lambda_{n}}\to 0, & \text{as}\;n\to \infty . \end{array}$$ (7)

Set *r_n_*=[*n*/*k_n_*] (the largest integer not exceeding *n*/*k_n_*) and put *J_ni_*=[0,*r_n_*/*n*]. Define the probability distribution π, on ${\mathbb{Z}}_{+}^{d_{1}}$ by

$$\pi_{\pi }\left(y\right)=P\left\{N_{n}\left(J_{\pi 1}\right)=y|N_{\pi }\left(J_{n1}\right)\neq 0\right\},y\in {\mathbb{Z}}_{+}^{dt}.$$

The following theorem which gives a useful characterization of the convergence of *N_n_* is an immediate consequence of the results in Sec. 5 of Ref. [12].

Theorem 2.1. *N_n_*→*^d^ N*_o_ *if and only if* π*_n_*→*^w^* π *and P* {*M_n_*≤*v_n_*(*t*)}→ e^−^*^v^*, *and in that case the Laplace Transform of N*_0_ *is given by* Eq. (6).

Next we consider the iid case in some detail and obtain an interesting connection with Theorem 5.3.1 of Ref. [8].

Proposition 2.2. *Let* {*ξ_n_*} *be iid and for fixed* **t**∈*T let N_n_ be defined by* Eq. (5). *If N_n_*→*^d^ N*_0_ *then the multiplicity distribution* π *in Eq. (6) is supported on the set S*−{0,1}*^d^*\{0}.

Proof: Observe that Δ(*v_n_*(t)) is trivially satisfied since *α_n_*_.1_=0 so that we may take *l_n_*≡1 and *k_n_=n*. Then ππ(y)=P{δ1=y|ξ1≤vκ(t)},y∈ℤ+d1, which is clearly supported on *S*. The result is now immediate since *S* is a closed set and π*_n_*→*^H^* π by Theorem 2.1.

Making the dependence on **t** explicit, we now write $N_{n}=N_{n}^{\left(t\right)}$, $N_{0}=N_{0}^{\left(t\right)}$, *v*=*v*^(t)^. In addition we shall require the following notation from Ref. [8]. For 1≤*k*≤*d*, let j(*k*)=(*j*_1_,…,*j_k_*) denote a vector with integer-valued components 1≤*j*_1_<*j*_2_<…<*j_k_*≤*d*, and for *x*=(*x*_1_,…,*x_d_*)∈IR*^d^* write *x_j_*_(_*_k_*_)_=(*x_j_*_1_,…,*x_jk_*). Define the “survival function”

$$G\left(x\right)=P\left\{\xi_{11}>x_{1},\ldots ,\xi_{1d}>x_{d}\right\}$$

and write $G_{j\left(k\right)}\left(x\right)=P\left\{\xi_{1j_{1}}>x_{j_{1}},\ldots ,\xi_{1j_{k}}>x_{j_{k}}\right\}$. For each **j**(*k*), let y_j(_*_k_*_)_ denote the element in *S*={0,1 }^d^\{0} whose *j*th component equals 1 if and only if *j*=*j_i_* for some *i*=1,…,*k*. (This defines a natural 1-1 correspondence between *S* and the **j**(*k*)’s.)

Theorem 2.3. *Let* {*ξ_n_*} *be iid. Then* $N_{0}^{\left(t\right)}\to^{d}N_{0}^{\left(l\right)}$ *for some fixed* **t**∈*T if and only if*

$$\underset{n\to \infty }{\lim}\;nG_{j\left(k\right)}\left(v_{n}\left(t\right)\right)=h_{j\left(k\right)}\left(t\right)<\infty$$ (8)

*for each* **j**(*k*).1≤*k*≤*d. In that case* $N_{0}^{\left(t\right)}$ *has Laplace Transform given by* Eq. (6) *with*

$$v^{\left(t\right)}=\sum_{k=1}^{d}{\left(-1\right)}^{k+1}\sum_{1\leq j<,\ldots <j_{k}\leq d}h_{j\left(k\right)}\left(t\right)$$ (9)

*and with* π^(t)^ *determined by the relations*

$$h_{j\left(k\right)}\left(t\right)=v^{\left(t\right)}\sum_{y\geq y_{j\left(k\right)}}\pi^{\left(t\right)}\left(y\right).$$ (10)

Proof: Write $S_{k}\left(t\right)=\sum_{1\leq j\;1<\ldots <j_{1}\leq d}G_{j\left(k\right)}\left(t\right)$, so that P{ξ1≤vn(t)}=∑k=1d(−1)k+1Sk(vn(t)). If Eq. (8) holds for each *k*, then

limn→∞nP{ξ1≤vn(t)}=∑k=1d(−1)k+1∑1≤j1<,…<jk<dhJ(k)(t)=v(t), (11)

and hence $\lim_{n\to \infty }P\left\{M_{R}\leq v_{n}\left(t\right)\right\}=e^{-v^{10}}$.

Next observe that for each j(*k*). 1≤*k*≤*d*,

Gj(k)(vπ(t))=∑y≥yj(k)P{δ1=y}=P{ξ1≤vπ(t)}∑y≥yj(k)πn(y), (12)

where πn{y)=P(δ1−y|ξ1≤vn(t)}. Moreover, this relationship is invertible in the sense that each of the probabilities π*_n_*(y), y∈*S*, can be expressed as a linear combination of the *G*_j(k)_(*v_n_*(**t**))’s. Therefore by Eq. (8). lim*_n_*_→∞t_π*_n_*(y)=π^(t)^(y) (say) exists and satisfies Eq. (10). Hence by Theorem 2.1 $N_{n}^{\left(t\right)}\to^{d}N_{0}^{\left(t\right)}$ where $N_{0}^{\left(t\right)}$ has the specified parameters. Conversely if $N_{n}^{\left(t\right)}\to^{d}N_{0}^{\left(t\right)}$ then π*_n_* and *P*{*M_n_*≤*v_n_*(**t**)} converge (by Theorem 2.1 again), and hence Eq. (8) follows by virtue of Eqs. (11) and (12) □.

Corollary 2.4. *Let* {*ξ_n_*} *be iid. Then* $N_{n}^{\left(t\right)}\to^{d}N_{0}^{\left(t\right)}$ *for each* **t**∈*T if and only if P*{*M_n_*≤*v_n_*(**t**)}→*^w^ H*(**t**). *Moreover H and* {*v*^(t)^,π^(t)^}_t∈_*_T_ determine each other*.

Proof: (Sketch) The first part follows from Theorem 2.3 above and Theorem 5.3.1 of Ref. [8] which states that *P*{*M_n_*≤*v_n_*(**t**)}→*^w^ H*(**t**) if and only if Eq. (8) holds for each **t**∈*T*. Note that *H*(t)=e^−^*^v^*^(t)^ so that *H* and the *v*^(t)^’*s* can be obtained from each other. Also the π^(t)^’*s* can be obtained from the *v*^(t)^’s by first inverting Eq. (9) to get the *h*_j(_*_k_*_)_(**t**)’s and then inverting Eq. (10). (The inversion of Eq. (9) is carried out inductively using the fact that the weak convergence of *H_n_*(*v_n_*(**t**)) implies that of all lower dimensional marginals.) □

Analogous results for the dependent case take on a slightly different form. Let {*ξ_n_*} be a stationary sequence satisfying Δ(*v_n_*(**t**)) for each **t**∈*T*. As before let *r*_R_=[*n*/*k_n_*] where {*k_n_*} is any sequence satisfying Eq. (7). and define

$$G_{r_{n}1\left(k\right)}\left(v_{n}\left(t\right)\right)=P\left\{M_{r_{n}j_{1}}>V_{\pi j_{1}}\left(t_{j1}\right),\ldots ,M_{r_{n}it}>v_{ji}\left(t_{ji}\right)\right\}.$$

Theorem 2.5. *Let* {*ξ_n_*}*be a stationary sequence satisfying* Δ(*v_n_*(*t*)) *for each* **t**∈*T. Then P*{*M_R_*≤*v_n_* (t))→^w^ *H*(**t**) *if and only if*

$$\underset{n\to \infty }{\lim}k_{\pi }G_{r_{n}j\left(k\right)}\left(v_{n}\left(t\right)\right)=h_{j\left(k\right)}\left(t\right)<\infty$$

*for each* **j**(*k*), 1≤*k*≤*d and* **t**∈*T*, *and in that case*

$$H\left(t\right)=\exp\left\{\sum_{k=1}^{d}{\left(-1\right)}^{k}\sum_{1<j_{1}<\cdots <j_{k}\leq d}h_{j\left(k\right)}\left(t\right)\right\}.$$

Proof: Observe that the mixing condition Δ(*v_n_*(**t**)) implies that {*ξ_n_*_j(_*_k_*_)_} satisfies Δ(*v_n_*_j(_*_k_*_)_(**t**)) for each **j**(*k*) (with obvious notation). Hence it may be shown as in the univariate case (see Lemma 2.1 of Ref. [1]) that

$$P\left\{M_{nj\left(k\right)}\leq v_{nj\left(k\right)}\left(t\right)\right\}-P^{k_{n}}\left\{M_{r_{n}j\left(k\right)}\leq v_{nj\left(k\right)}\left(t\right)\right\}\to 0.$$ (13)

for each **j**(*k*). The result may therefore be proved in exactly the same way as Theorem 5.3.1 of Ref. [8]. □

Remark: Under the hypothesis of Theorem 2.5, if $N_{n}^{\left(t\right)}\to^{d}N_{0}^{\left(t\right)}$, **t**∈*T*, with parameters *v*^(t)^ and π^(t)^ then $P\left\{M_{n}\leq v_{n}\left(t\right)\right\}\to^{w}\;H\left(t\right)-c^{-\rho^{\left(t\right)}}$, as in the iid case. However it is not possible in general to recover the π^(t)^’s from *H* since the clustering of exceedances may cause the support of π^(t)^ to extend beyond *S*. References [9, 10] give sufficient conditions (analogous to the *D*^1^(*u_n_*) condition of Ref. [13]) under which clustering docs not occur, so that Corollary 2.4 can be extended to stationary sequences satisfying this condition.

A distribution function *F* on IR*^d^* is said to be *independent* if $F\left(x\right)=\prod_{j=1}^{d}F_{j}\left(x_{j}\right),\;x\in {\text{IR}}^{d}$. If {*ξ_n_*} is iid and P{*M_n_*≤*v_n_*(*t*)}→*^w^ H*(t), then it follows from Corollary 5.3.1 of Ref. [8] that *H* is independent if and only if the marginals of *H* are pairwise independent. The analogous result for the dependent case is stated below. The proof (which is omitted) is essentially the sanie as for the iid case, but uses Theorem 2.5 instead of Theorem 5.3.1 of Ref. [8].

Corollary 2.6. *Let* {*ξ_n_*} *be a stationary sequence satisfying* Δ(*v_n_*(t)) *for each* **t**∈*T and suppose that P* {*M_n_*≤*v_n_*(**t**)}→*^w^ H*(**t**). *Then H is independent if and only if* $k_{n}P\left\{M_{r_{n}j}>v_{n}j\left(t_{j}\right),\;M_{r_{n}-1}>v_{nJ}\left(t_{1}\right)\right\}\to 0$ *for each* 1≤*j*<*l*≤*d*, **t**∈*T*, *i.e.*, *if and only if* $k_{n}G_{r_{n}j\left(k\right)}\left(v_{n}\left(t\right)\right)\to 0$ *for each* **j**(2) *and each* **t**∈*T*.

It is shown in Ref. [14] that *H* is independent it $H\left(t\right)=\prod_{j-1}^{d}H_{j}\left(t_{j}\right)$ for some **t**∈(0,1)*^d^*. Although the result in [14] only stated for iid sequences under a linear normalization, the proof essentially rests on the defining property of extreme dependence functions, namely Eq. (1). Consequently the result extends lo the present more general situation allowing dependence and non-lineal normalizations. Corollary 2.6 can therefore be improved as follows.

Corollary 2.7. *Let* {*ξ_n_*} *be as in Corollary 2.6 and suppose that P*{*M_n_*≤*v_n_*(**t**)}→*^w^ H*(**t**).* Then the following are equivalent:*

- *H is independent*,
- $H\left(t\right)=\prod_{j-1}^{d}H_{j}\left(t_{j}\right)$*for some***t**∈(0,1)*^d^*,
- $k_{n}G_{r_{n}j\left(k\right)}\left(v_{R}\left(t\right)\right)\to 0$*for each* j(2).*for some* **t**∈(0,1)*^d^*.

It should be noted that Refs. [9, 10] give some interesting sufficient conditions for *H* to be independent when {*ξ_n_*} is a stationary sequence. A natural question to ask in the present context is whether *H* is independent whenever *Ĥ* is. Proposition 3.4 gives a necessary and sufficient condition for this in terms of the extremal index, but the answer in general is negative and a counter-example can be found in [10]. It seems more plausible that the converse may be true, i.e., that *Ĥ* is independent whenever *H* is. In fact however, this too is not the case, as shown by an interesting counter-example in [15].

We conclude this section by stating a result which extends Theorem 5.1 of [5] and is proved similarly.

Theorem 2.8. *Let* {*ξ_n_*} *be a stationary sequence satisfying* Δ(*v_n_*(**t**)) *for each* **t**∈*T and suppose that* $N_{n}^{\left(t\right)}\to^{d}N_{0}^{\left(t\right)}$ *for some* **t**∈*T. Then* $N_{n}^{\left(t^{c}\right)}\to^{d}N_{0}^{\left(\tau^{d}\right)}$ *for each c*>0 *and fur thermore*, $v^{\left(t^{c}\right)}=cv^{\left(t\right)}$ *and* $\pi^{\left(t^{c}\right)}=\pi^{\left(t\right)}$ (*where* $t^{c}=\left(t_{1}^{c},\ldots ,t_{d}^{c}\right)$).

## 3. The Multivariate Extremal Index

Let {*ξ_n_*} be a stationary sequence and $\left\{{\hat{\xi }}_{n}\right\}$ the associated iid sequence. Suppose that *P*{*M_n_*≤*v_n_*(*t*)}*→^w^ H*(**t**) and $P\left\{{\hat{M}}_{n}\leq v_{n}\left(t\right)\right\}\to^{w}\hat{H}\left(t\right)$. The *multivariate extremal index* of {*ξ_n_*} is then defined by the relation *H*(**t**)−*Ĥ^θ^*^(**t**)^(**t**) (see Eq. (1)), or more explicitly

$$0\left(t\right)-\log H\left(t\right)/\log\;\hat{H}\left(t\right),\;t\in T.$$

Obscene that *θ*(**t**) is well defined since *Ĥ* has Uniform[0,1] marginals and hence, 0<*Ĥ*(**t**)<1 on *T*. The following results describe some basic properties of the multivariate extremal index.

Proposition 3.1. *Assume that* {*ξ_n_*}*satisfies* Δ(*v_n_*(**t**)) *for each* **t**∈*T and has extremal index θ*(**t**). *Then*

- *θ*(**t**)=*θ*(t*^c^*) *for each***t**∈*T and c*>0, *and*
- *for each j*=1,…,*d*, {*ξ_nj_*} *has extremal index θ_j_*=*θ*(**t**) *where***t**∈*T has all coordinates equal to I except the jth*.

(Note that by (*i*), *θ*(**t**) is constant along the contours *L*_1_={t*^c^*:*c*>0}, **t**∈*T*, and hence *θj* in (*ii*) is well defined.)

Proof: Recall mat (by Theorem 1.1) *H*(t*^c^*)=*H^c^*(t) and *Ĥ*(**t***^c^*)=*Ĥ^c^*(**t**) so that (*i*) follows from the definition of the extremal index. Next, for **t**∈*T* with all coordinates but the *j*th equal to 1, *P*{*M_n_*≤*v_n_*(**t**)}=*P*{*M_nj_*≤*v_nj_*(t*_j_*)} and hence

$$\underset{\pi \to \infty }{\lim}P\left\{M_{nj}\leq v_{nj}\left(l_{j}\right)\right\}=\underset{\pi \to \infty }{\lim}P\left\{M_{n}\leq v_{n}\left(t\right)\right\}=H\left(t\right)=H_{j}\left(t_{j}\right).$$

Therefore by Theorem 2.2 of Ref. [1], {*ξ_nj_*} has extremal index *θ_j_* (say) so that $H_{j}\left(t_{j}\right)=t_{J}^{\theta_{j}}$. Now *H*(**t**)−*Ĥ^θ^*^(t)^(**t**) by definition of the extremal index, and for the present choice of **t** this is the same as $H_{j}\left(t_{j}\right)=t_{j}^{\theta \left(t\right)}$, whence it follows that *θ*(**t**)=*θ_j_* for all such **t**.

For **t**∈*T*, let ${\bar{N}}_{n}^{\left(t\right)}$ denote the one-dimensional point process obtained from $N_{\pi }^{\left(t\right)}$ via the map **y**→*l*_(**y**≠0)_ from {0,1}*^d^* to {0,1}, i.c., ${\bar{N}}_{\pi }^{\left(t\right)}\left(B\right)-\sum_{i-1}^{n}I_{\left(i^{\prime }n\in B\right)}I_{\left(\delta_{i}\neq \cap \right)}$, *B*∈*ℬ*. Thus ${\hat{N}}_{n}^{\left(t\right)}$has unit mass at *i*/*n* if and only if *ξ*_i_≰*v_n_*(t). Assume that {*ξ_n_*} satisfies Δ(*v_n_*(**t**)) and with J*_n_*_l_ as in Sec. 2. let

$${\bar{\pi }}_{n}^{\left(t\right)}\left(y\right)=P\left\{N_{n}^{\left(t\right)}\left(J_{n1}\right)=y|{\bar{N}}_{n}^{\left(t\right)}\left(J_{n1}\right)>0\right\}.\;y\geq 1.$$

Proposition 3.2. *Assume that* {*ξ_n_*}*satisfies* Δ(*v_n_*(**t**)) *for each* **t**∈*T and has extremal index θ*(**t**). *Then* $\theta \left(t\right)={\left(\lim_{\alpha \to \infty }\;\sum_{y>1}y\pi_{n}^{-\left(t\right)}\left(y\right)\right)}^{-1}$.

Proof: Observe that

∑y>1yπn(t)(y)=E(N˜n(t)(Jn1)|N˜n(t)(Jn1)>0)rnP{ξ1≤vn(t)}P{N˜n(t)(Jn1)>0}=knrnnnP{ξ1≤vn(t)}knP{Mrn≤vn(t)}.

Now $\lim_{n\to \infty }\;P\left\{{\bar{M}}_{n}\leq v_{n}\left(t\right)\right\}=\hat{H}\left(t\right)$ and lim*_n_*_→∞_ *P*{*M_n_*≤*v_n_*(**t**)}=*H*(**t**) (by assumption), so that lim*_n_*_→∞_ *nP*{*ξ*_l_≰*v_n_*(**t**)}=−log *Ĥ*(**t**) and (by Eq. (13)) limn→∞knP{Mrn≤vn(t)}=−logH(t). Therefore $\lim_{n\to \infty }\;\sum_{y\geq 1}\;y{\tilde{\pi }}_{n}^{\left(t\right)}(y)=\log\;\hat{H}\left(t\right)/\log\;H\left(t\right)-1/\theta \left(t\right)$, as required. □

Remark: Proposition 3.2 is simply the multivariate version of Eq. (1) and shows how the extremal index is related to the clustering of “exceedances.” Indeed, according to the present viewpoint, an exceedance occurs at time *i* if *ξ*≰*v_n_*(**t**), i.c., if *ξ_nj_*>*v_nj_*(*t_j_*) for at least one *j*. Thus Propositions 3.1 and 3.2 show that while the degree of clustering may depend on **t**, it is constant on each *L*_t_. Note also the connection to Theorem 2.8.

The next result gives the relation between the dependence functions of *H* and *Ĥ*, which is seen to involve the extremal index in an intricate manner.

Proposition 3.3. *If* {*ξ_n_*} *has extremal index θ*(**t**), **t**∈*T. then*

$$D_{H}\left(t_{1}^{\theta_{1}},\ldots ,t_{d}^{\theta_{d}}\right)=D_{H}^{\theta \left(t\right)}\left(t\right),\;t\in T,$$ (14)

*where θ_j_ is the extremal index of* {*ξ_n_*}, *j*=1,…,*d*.

Proof: By definition of the dependence function, *D_H_*(**t**)=*P*{*H*_l_(*X*_l_)≤*t*_l_,…,*H_d_*(*X_d_*)≤*t_d_*} where (*X*_l_,…, *X_d_*) ts a random vector with distribution *H*. Therefore, since $H_{j}\left(t_{j}\right)=t_{j}^{\theta_{j}}$.

$$\begin{array}{c} D_{H}\left(t_{1}^{\theta_{1}},\ldots ,t_{d}^{\theta_{d}}\right)=\left\{X_{1}\leq t_{1},\ldots ,X_{d}\leq t_{d}\right\}=H\left(t\right)={\hat{H}}^{\left(\theta \right)}\left(t\right)\\ =D_{H}^{\left(\theta \right)}\left(t\right), \end{array}$$

or required. □

Remarks

1. Note that s=t*^c^* (for some *c*>0) if and only if log s*_j_*/log *s_d_*=log *t_j_*/log *t_d_*−*a_j_* (say), *j*=1,*…*,*d* − 1. Therefore we may write *L*_t_=*L*_a_ where a=(*a*_1_,…,*a_d_*_−1_), and hence by the remark following Proposition 3.2, *θ*(**t**)=*θ*(**a**), i.c., the extremal index is a function of *d* − 1 variables only.
2. By Proposition 3.3, $D_{H}\left(t_{1}^{\theta_{1}},\ldots ,t_{d}^{\theta_{d}}\right)=D_{H}^{\theta \left(t\right)}\left(t\right)-D_{H}\left(t^{\theta \left(t\right)}\right)$. Also, if **t**∈*L*_a_ then $\left(t_{1}^{\theta_{1}},\ldots ,t_{d}^{\theta_{d}}\right)\in L_{a*}$, where a*=(*a*_1_*θ*_1_/*θ_d_*,…,*a_a_*_−1_*θ_d_*_−1_/*θ_d_*). Thus *D_H_* is obtained by translating the values of *D_Ĥ_*(=*Ĥ*) on *L*_a_ onto $L_{a^{*}}$.
3. While the above results illustrate some of the basic properties of the multivariate extremal index, they are far from complete. For instance; it would be useful to identify the set of all “admissible” *θ*(•) for a given *Ĥ*, that is the set of all *θ*(•) such that *D_H_*(•) defined by Eq. (14) is a probability distribution on [0,1]*^d^*. It would also be of interest to study the properties of *θ*(•) when one or both of *Ĥ* and *H* are independent. In this context we have the following simple result.

Proposition 3.4. *If Ĥ is independent*, *then Ĥ is independent if and only if*

$$\theta \left(t\right)=\sum_{j=1}^{d}\theta_{j}\log t_{j}/\sum_{j=1}^{d}\log t_{j},\;for\;some\;t\in {\left(0,1\right)}^{d},$$

*In particular, if both Ĥ and H are independent then θ*(**t**) *is a convex combination of the θ_j_’s*.

Proof: If *Ĥ* is independent, then $H\left(t\right)-\hat{H}{\left(t\right)}^{\theta \left(t\right)}={\left(\prod_{j-1}^{d}I_{j}\right)}^{\theta \left(t\right)}$. The conclusion follows immediately from Corollary 2.7 (iii) upon taking logarithms and noting that if *H* is independent, then $H\left(t\right)=\prod_{j-1}^{d}t_{j}^{\theta j}$.

The extremal index can be given the following more general formulation. Let $\hat{\mu }$and *μ* be the probability measures on (0,1)*^d^* corresponding to *Ĥ* and *H*, respectively. Thus for instance,

$$\mu \left(A\right)=\underset{n\to \infty }{\lim}P\left\{M_{n}\in v_{n}\left(A\right)\right\}$$

where *v_n_*(*A*)={*v_n_*(s) : s∈*A*}, *A*⊂(0,1))*^d^*. We now define $\bar{\theta }\left(A\right)$ via the relationship $\mu \left(A\right)={\hat{\mu }}^{\theta \left(A\right)}\left(A\right)$, or more directly $\bar{\theta }\left(A\right)=\log\mu \left(A\right)/\log\hat{\mu }\left(A\right)$, for subsets *A*⊂(0,1)*^d^* such that $\hat{\mu }\left(A\right)>0$ and *μ*(*A*)>0.

Note that $\theta \left(t\right)-\bar{\theta }\left(\left(0,t_{1}\right)\times \ldots \times \left(0,t_{d}\right)\right)$for **t**∈*T*. Thus if {*θ*(**t**): **t**∈*T*} is known along with either of *H* or *Ĥ*, then it is possible at least in theory to obtain $\left\{\bar{\theta }\left(A\right):A\subset {\left(0,1\right)}^{d}\right\}$. In practice, however, it may not be possible to obtain $\bar{\theta }\left(A\right)$in a tractable form, but frequently one is only interested in certain special sets, typically rectangles of the form $\prod_{j-1}^{d}\left(a_{j},b_{j}\right)$, and for such sets the computation is easy.

The definition of *M_n_* as the vector of component wise maxima actually corresponds to regarding *ξ_i_* as an extreme observation if *ξ_ij_*>*v_nJ_*(*t_j_*) for some *j*. More generally, one may define *ξ_i_* to be an extreme value if *ξ_i_∈v_n_*(A) for some *A*⊂(0,1)*^d^*, in which case $\bar{\theta }\left(A\right)$ has an interpretation as a measure of the clustering of such extremes. Note that the original definition of extremes corresponds to letting *A*=((0,*t*_1_)×…×(0,*t*_d_)*^c^*. Alternately one may consider taking *A*−(*t*_1_,l)×…×(*t_d_*,l) which corresponds to defining *ξ*_1_ as an extreme observation if *ξ_ij_*>*v_nj_*(*t_j_*) for all *j*. Yet another choice is $A=\left\{t:\Sigma t_{j}^{2}>c\right\}$.

## 4. Examples

We conclude with two examples, both involving bivariate stationary sequences.

Example 4.1 Let {*η_n_*} be an iid sequence, and put *ξ_n_*_1_=*η_a_*, and *ξ_n_*_2_=max{*η_n_*_−1_, *η_n_*}. Let *F* denote the distribution of *ξ_n_*=(*ξ_n_*_1_, *ξ_n_*_2_) with marginals *F*_1_ and *F*_2_. Then $F_{2}\left(x\right)=P\left\{\xi_{n2}\leq x\right\}=P\left\{\eta_{n-1}\leq x,\;\eta_{n}\leq x\right\}=F_{1}^{2}\left(x\right)$ and

$$F\left(x_{1},x_{2}\right)=P\left\{\xi_{n1}\leq x_{1},\xi_{n2}\leq x_{2}\right\}=\{\begin{array}{l} \begin{array}{cc} F_{1}^{2}\left(x_{2}\right), & \;\text{if}\;x_{1}\geq x_{2} \end{array}\\ \begin{array}{cc} F_{1}\left(x_{1}\right)F_{1}\left(x_{2}\right), & \text{if}\;x_{1}<x_{2.} \end{array} \end{array}$$

If *v_nj_*(*t_j_*) satisfies $F_{j}^{n}\left(v_{nj}\left(t_{j}\right)\right)\to t_{j},\;j=1,2$ then $\lim_{n\to \infty }F_{1}^{n}\left(v_{n2}\left(t_{2}\right)\right)=t_{2}^{1/2}$ so that $v_{n2}\left(t_{2}\right)=v_{n1}\left(t_{2}^{1/2}\right)$. Moreover *v_n_*_1_(*t*_1_)≥*v_n_*_2_(*t*_2_) if and only if $t_{1}\geq t_{2}^{1/2}$, and so

$$H_{n}\left(v_{n}\left(t\right)\right)=P\left\{M_{n}\leq v_{n}\left(t\right)\right\}\to^{n}H\left(t\right)=\{\begin{array}{ll} t_{2}^{1/2}, & \text{if}\;t_{1}\geq t_{2}^{1/2}\\ t_{1,} & \text{if}\;t_{1}<t_{2}^{1/2}. \end{array}$$

The marginals of *H* are therefore *H*_1_(*t*_1_)=*t*_1_ and $H_{2}\left(t_{2}\right)=t_{2}^{1/2}$ so that *θ*_1_=1 and *θ*_2_=1/2, and the dependence function of *H* is DH(t)=H(t1,t22)=t1^t2. For the associated iid sequence $\left\{{\hat{\xi }}_{n}\right\}$ on the other hand, it is easily verified that

$${\hat{H}}_{n}\left(v_{n}\left(t\right)\right)=P\left\{{\hat{M}}_{n}\leq v_{n}\left(r\right)\right\}\to \hat{H}\left(t\right)=\{\begin{array}{ll} t_{2}, & \text{if}\;r_{1}\geq t_{2}^{1/2}\\ r_{1}t_{2}^{1/2}, & \text{if}\;t_{1}<t_{2}^{1/2}. \end{array}$$

from which it follows that

$$\theta \left(t\right)=\{\begin{array}{ll} \frac{1}{2t} & \text{if}\;t_{1}\geq t_{2}^{1/2}\\ \frac{\log\;t_{1}}{\log t_{1}t_{2}^{1}} & \text{if}\;t_{1}<t_{2}^{1/2}. \end{array}$$

We next consider a moving average sequence studied in Ref. [16].

Example 4.2, Let {Z*_k_*=(Z*_k_*_1_,Z*_k_*_2_)^1^}, −∞<*k*<∞, be a sequence of iid random vectors in IR^2^. We assume the existence of a sequence of positive constants *a_n_*→∞, and a measure *v* on IR^2^ which is finite on sets of the form {x : ‖x‖>r}, r>0 (where ‖⋅‖ denotes the Euclidean norm in IR^2^), such that *nP*{*a_n_* ^1^Z_0_∈•}→*^v^ v*(•). (Here ‘→*^v^*’ denotes vague convergence of measures on IR^2^ with respect to the metric $d\left(x_{1},x_{2}\right)=\left|r_{1}^{-1}-r_{2}^{-1}\right|\vee \left|\theta_{1}-\theta_{2}\right|$, where for *i*=1,2,*r*, and *θ_i_* denote the polar coordinates of x*_j_*, and *a*∨*b*=max{*a*,*b*}.) The measure *v* is necessarily of the form *v*({**x** : ‖**x**‖>*r*,*θ*(**x**)∈A})=*r*^−^*^a^S*(A) for *r*>0 and *A*⊂(0,2π), where *S*(•) is a probability measure on (0,2π) and *α*>0. Hence in particular [17].

$$v\left(cA\right)=c^{-\sigma }v\left(A\right)$$ (15)

for all *c*>0 and all sets *A* with *v*(*A*)<*∞*.

Define the bivariate moving average process $X_{n}=\sum_{j-n}^{\infty }C_{j}Z_{n}{\;}_{j}$, where ${\left\{C_{j}={\left[c_{jkl}\right]}_{k\;J-1}^{2}\right\}}_{j\geq 0}$ is a sequence of real 2×2 matrices satisfying $\sum_{j=0}^{\infty }{\left|C_{jkl}\right|}^{\delta }<\infty ,\;k,\;l=1,\;2$, for some δ∈(0,*α*). δ≤1. For x=(x_1_,x_2_)′∈IR^2^, write *A_xj_*= {z : C*_j_*z∈((−∞,*x*_1_)×(−∞,*x*_2_))*^c^*}, where A*^c^* denotes the complement of a set A⊂IR^2^. Then [16].

$$\begin{array}{c} \underset{x\to \infty }{\lim}P\left\{a_{n}^{-1}{\hat{M}}_{n}\leq x\right\}=\exp\left\{-\hat{\gamma }\left(x\right)\right\},\text{and}\\ \underset{x\to \infty }{\lim}P\left\{a_{n}^{-1}M_{n}\leq x\right\}=\exp\left\{-\gamma \left(x\right)\right\},x\in {\mathrm{IR}}^{2}, \end{array}$$

where $\hat{\gamma }\left(x\right)=\sum_{j=0}^{\infty }v\left(A_{xj}\right)$ and $\gamma \left(x\right)=v\left(\cup_{j-0}^{\infty }A_{xj}\right)$. The extremal index is therefore $\theta \left(x\right)=\gamma \left(x\right)/\hat{\gamma }\left(x\right),\;x\in {\mathrm{IR}}^{2}$. It follows from the definition of *A_xj_* and Eq. (15) that this is in fact a function of *x*_1_*/x*_2_. Note that the extremal index defined above differs from that in Sec. 3 in that it is defined on IR^2^ rather than [0,1]^2^. However the two definitions arc equivalent as may be seen by means of a suitable transformation from IR^2^ to [0,1]^2^.

The actual calculation of *θ*(**x**) may be quite difficult in general, but possible to carry out under appropriate simplifying assumptions.

*Case (i)*. If *C_j_*=*c_j_C* where $C={\left[C_{kt}\right]}_{kj-1}^{2}$, and the *c_j_*’s are non-negative constants, then $A_{x_{J}}=C_{J}^{-1}B\left(x\right)$ and $\cup_{j=0}^{\infty }A_{x_{J}}=cB\left(x\right)$, where *B*(x)={z : *C*z∈((−∞,*x*_1_)× (−∞,*x*_2_))*^c^*} and *c*=max{*c_j_* : *j*≥0}. Therefore by Eq. (15), $v\left(A_{xj}\right)=c_{j}^{\alpha }v\left(B\left(x\right)\right)$ and $v\left(\cup_{j=0}^{\infty }A_{x\;j}\right)=c^{\alpha }v\left(B\left(x\right)\right)$ so that $\theta \left(x\right)\equiv c^{\sigma }/\sum_{j=0}^{\infty }c_{j}^{\alpha }$.

*Case (ii)*. If the *C_j_*’s are diagonal, i.e., *C_j_*=diag[*C_j_*_1_,*C_j_*_2_] with *c_ji_*≥0, *i*=1,2, then *A_xj_*={z : *c_ji_z*_1_>*x*_1_ or *c_j_*_2_*z*_2_>*x*_2_} and $\cup_{j=0}^{\infty }A_{x\;j}={\left(\left(-\infty ,\;x_{1}/c_{1}\right)\times \left(-\infty x_{2}/c_{2}\right)\right)}^{c}$where *c_i_*= max{*c_ji_* : *j*≥0}, *i*=1,2. In particular, taking *x*_2_=∞ and using Eq. (15) as in Case (i), we have $v\left(A_{xj}\right)=c_{ji}^{\alpha }v\left(\left\{z:z_{1}>x_{1}\right\}\right)$ and $v\left(\cup_{j=0}^{\infty }A_{xj}\right)=c_{1}^{\alpha }v\left(\left\{z:z_{1}>x_{1}\right\}\right)$, so that the extremal index of $\theta_{1}=c_{1}^{\alpha }/\sum_{j=0}^{\infty }C_{J-1}^{\alpha }$.

*Case (iii)*. Let *D* denote the support of *v*. If *D*⊂ {z : *z*_1_=0 or *z*_2_=0} (which is the case if the coordinates of **Z**_0_ are independent), then we may write

$$v{\left(\left(-\infty ,x_{1}\right)\times \left(-\infty ,x_{2}\right)\right)}^{c}=a_{1}x_{1}^{-\alpha }+a_{2}{x_{2}}^{-\alpha },\;x_{1},x_{2}\geq 0,$$ (16)

for suitable constants *a*_1_≥0 and *a*_2_≥0. Once again, assuming the *c_jkl_*’s to be non-negative and writing *c_ki_*=max{*c_jki_* : *j*≥0} for *k*,*l*=1,2, we have (writing *a*∧*b*=min{*a*,*b*})

$$A_{1j}\cap D={\left(\left(-\infty ,x_{1}/c_{j,11}\wedge x_{2}/c_{j,21}\right)\times \left(-\infty ,x_{1}/c_{j,12}\wedge x_{2}/c_{j,22}\right)\right)}^{c}\cap D$$

and

$$\cup_{j=0}^{\infty }A_{x,j}\cap D={\left(\left(-\infty ,x_{1}/c_{11}\wedge x_{2}/c_{21}\right)\times \left(-\infty ,x_{1}/c_{12}\wedge x_{2}/c_{22}\right)\right)}^{c}\cap D.$$

so that using Eq. (16)

$$\theta (x)=\frac{a_{1}{\left(x_{1}/c_{11}\wedge x_{2}/c_{21}\right)}^{-\alpha }+a_{2}{\left(x_{1}/c_{12}\wedge x_{2}/c_{22}\right)}^{-\alpha }}{a_{1}\sum_{j=0}^{\infty }{\left(x_{1}/c_{j,11}\wedge x_{2}/c_{j,21}\right)}^{-\alpha }+a_{2}\sum_{j=0}^{\infty }{\left(x_{1}/c_{j,12}\wedge x_{2}/c_{j,22}\right)}^{-\alpha }}$$

Thus putting *x*_2_=∞, we have $\theta_{1}=\left(a_{1}c_{11}^{\alpha }+a_{2}c_{12}^{\alpha }\right)/\left(a_{1}\sum_{j=0}^{\infty }c_{j,11}^{\alpha }+a_{2}\sum_{j=0}^{\infty }c_{j,12}^{\alpha }\right)$ and similarly, $\theta_{2}=\left(a_{1}c_{21}^{\alpha }+a_{2}c_{22}^{\alpha }\right)/\left(a_{1}\sum_{j=0}^{\infty }c_{j,21}^{\alpha }+a_{2}\sum_{j=0}^{\infty }c_{j,22}^{\alpha }\right)$.

If also *c_j_*_,12_=*c_j_*_,12_=0 for each *j*, (that is if the *C_j_*’s are diagonal), then

$$\theta (x)=\frac{a_{1}x_{1}^{-\alpha }c_{11}^{\alpha }+a_{2}x_{2}^{-\alpha }c_{22}^{\alpha }}{a_{1}x_{1}^{-\alpha }\sum_{j=0}^{\alpha }c_{j,11}^{\alpha }+a_{2}x_{2}^{-\alpha }\sum_{j=0}^{\alpha }c_{j,22}^{\alpha }},$$

and in particular, $\theta_{1}=c_{11}^{\alpha }/\sum_{j=1}^{\infty }c_{j,11}^{\alpha }$ and $\theta_{2}=c_{22}^{\alpha }/\sum_{j=1}^{\infty }c_{j,22}^{\alpha }$. Note that in this case the limiting distributions of *M_n_* and ${\hat{M}}_{n}$ are both independent, and hence (in accordance with Proposition 3.4) *θ*(**x**) is a convex combination of *θ*_1_ and *θ*_2_.

The non-negativeness of the *C_j_*’s assumed abive is not crucial and may be related, although at the cost of more involved calculations.
